# Increased presence of nuclear DNAJA3 and upregulation of cytosolic STAT1 and of nucleic acid sensors trigger innate immunity in the ClpP-null mouse

**DOI:** 10.1007/s10048-021-00657-2

**Published:** 2021-08-03

**Authors:** Antonia Maletzko, Jana Key, Ilka Wittig, Suzana Gispert, Gabriele Koepf, Júlia Canet-Pons, Sylvia Torres-Odio, A. Phillip West, Georg Auburger

**Affiliations:** 1 Experimental Neurology, Medical School, Goethe University, 60590 Frankfurt, Germany; 2 Faculty of Biosciences, Goethe University, Altenhöferallee 1, 60438 Frankfurt, Germany; 3 Functional Proteomics, Faculty of Medicine, Goethe University, 60590 Frankfurt, Germany; 4 Department of Microbial Pathogenesis and Immunology, College of Medicine, Texas A&M, University Health Science Center, Bryan, TX 77807, USA

**Keywords:** PRLTS3, Release of mtDNA and mtRNA, cGAS-STING, Leukodystrophy, Ataxia, Mitochondrial amino acid tRNA synthetases, TWINKLE, POLG, MTRNR1

## Abstract

Mitochondrial dysfunction may activate innate immunity, e.g. upon abnormal handling of mitochondrial DNA in TFAM mutants or in altered mitophagy. Recent reports showed that also deletion of mitochondrial matrix peptidase ClpP in mice triggers transcriptional upregulation of inflammatory factors. Here, we studied ClpP-null mouse brain at two ages and mouse embryonal fibroblasts, to identify which signaling pathways are responsible, employing mass spectrometry, subcellular fractionation, immunoblots, and reverse transcriptase polymerase chain reaction. Several mitochondrial unfolded protein response factors showed accumulation and altered migration in blue-native gels, prominently the co-chaperone DNAJA3. Its mitochondrial dysregulation increased also its extra-mitochondrial abundance in the nucleus, a relevant observation given that DNAJA3 modulates innate immunity. Similar observations were made for STAT1, a putative DNAJA3 interactor. Elevated expression was observed not only for the transcription factors *Stat1/2*, but also for two interferon-stimulated genes (*Ifi44*, *Gbp3*). Inflammatory responses were strongest for the RLR pattern recognition receptors (*Ddx58*, *Ifih1*, *Oasl2*, *Trim25*) and several cytosolic nucleic acid sensors (*Ifit1*, *Ifit3*, *Oas1b*, *Ifi204*, *Mnda*). The consistent dysregulation of these factors from an early age might influence also human Perrault syndrome, where ClpP loss-of-function leads to early infertility and deafness, with subsequent widespread neurodegeneration.

## Introduction

In the human organism, mutations in the mitochondrial matrix peptidase ClpP lead to Perrault syndrome type 3 (PRLTS3). This syndrome was initially described as autosomal recessive premature ovarian failure combined with sensorineural hearing loss. Later, it was found to be accompanied by a growth deficit and a generalized neurodegenerative process with leukodystrophy upon neuroimaging. This progressive atrophy manifests as late-onset progressive ataxia and neuropathy [1–5]. ClpP is highly conserved from bacteria to mammals, playing a crucial role in mitochondrial responses to unfolded protein stress (UPR^mt^). It cooperates with the disaggregases ClpX and ClpB, chaperones of the Hsp70 and Hsp60 family, and co-chaperones of the DnaJ and the GrpE family [6]. Overall, ClpP deficiency leads to problems for the assembly of proteins, firstly during their interaction with RNA in mitoribosomes and secondly during their association with DNA in mitochondrial nucleoids [7–9]. In ClpP-null mice, the quantification of mtDNA (mitochondrial DNA) with expected full-length size via Southern blotting did not show any abnormal dosage. However, the quantification of short fragments of mtDNA via PCR in two independent labs detected a several-fold increase [3, 8, 10]. Jointly, these findings suggest the presence of double-strand breaks in the mtDNA of ClpP-null cells, due to improper assembly into supercoils. This mitochondrial pathology triggers innate immunity activation in the eukaryotic host cell to a degree, where altered skin microbiome defenses can modify the lifespan of ClpP-null mice [10, 11]. Mechanistically, it was demonstrated that the abnormal mtDNA of ClpP-null cells activates cGAS-STING signaling and modulates type I interferon release. It is known that dsDNA breaks in the mitochondrial nucleoids elicit the cGAS-STING signals and the immune responses via the STAT1 pathway [9, 12]. Thus, these downstream effects might occur also in ClpP mutants and are assessed in the present study.

Other genetic causes of Perrault syndrome include mutations in the mitoribosome chaperone ERAL1, the mitochondrial amino acid tRNA transferases HARS2 and LARS2, and the mitochondrial translation factor RMND1, highlighting the importance of mitoribosomal translation for fertility and neurodegeneration. Additional causes are mutations in the mitochondrial DNA helicase-primase TWNK and the mitochondrial transcription factor TFAM, emphasizing the relevance of mtDNA disassembly. Perrault syndrome was also reported to be caused by mutations in the peroxisomal factors HSD17B4 and PEX6. Also, mutations in GGPS1 can be responsible, an enzyme for the prenylation of proteins, which was observed to act as a determinant of UPR^mt^ [9, 13–15]. Furthermore, maternally inherited mutations in the mitochondrial DNA are known to cause progressive deafness via altered mitoribosome functions, and the chronic administration of aminoglycoside antibiotics can also lead to hearing deficits, presumably via their impact on mitoribosomal translation fidelity [16–18]. Overall, mitochondrial translation and transcription pathology appear to be the common denominator of Perrault syndrome pathogenesis.

**Fig. 1:**
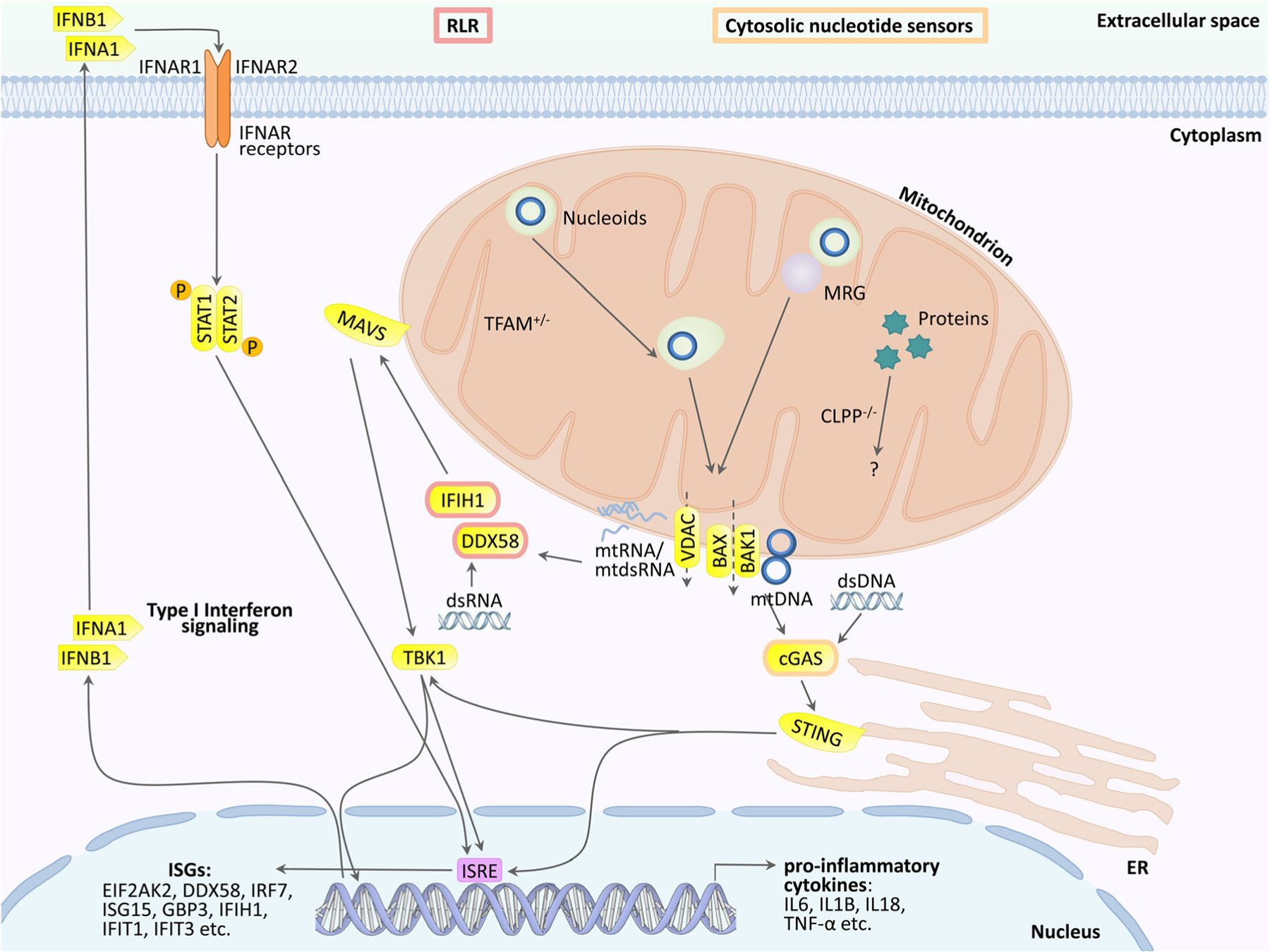
Innate immunity activation due to mutations in mitochondrial factors is mediated in the cytosol via the cGAS/STING pathway (when accumulated and possibly fragmented mtDNA is extruded to the cytosol) or the DDX58/IFIH1/MAVS sensors in the pattern recognition RLR pathway (when accumulated mitochondrial double-strand RNA is extruded). They stimulate the nuclear induction of interferon type I signaling. It is unclear if these established mechanisms also are prominent in ClpP-deficient cells, or additional pattern recognition receptors (TLR, NLR) and other cytosolic sensors play a relevant role. MRG, mitochondrial ribosomal granule; VDAC, PORIN; DDX58, RIG-I; IFIH1, MDA-5; STING, STING1, also known as TMEM173; ISRE, interferon-stimulated response element in the promoter of nuclear genes; other symbols can be retrieved in the GeneCards database

Particularly for TFAM mutations, the mechanism of innate immune activation has been elucidated in detail. Heterozygous TFAM deficiency results in abnormal packaging of mtDNA nucleoids and their extrusion into the cytosol, where TFAM-associated U-turn DNA will nucleate cGAS dimers and activate STING/TBK1. Extra-mitochondrially, any damage-associated molecular pattern (DAMP) that bears similarity to bacterial or viral components will trigger a signaling cascade via DDX58=RIG-I/IFIH1=MDA5/MAVS/TBK1. Ultimately, this cascade upregulates interferon-stimulated gene expression (see schematic overview in Figure 1). These responses mediate resistance against microbial infections and enhance acute repair mechanisms that are protective for the nuclear genome long-term stability [19–25].

Not only the rare Perrault syndrome shows such a combination of a primary mitochondrial dysfunction with subsequent sterile inflammation, but also the frequent neurodegenerative process in Parkinson’s disease (PD) has this dual characteristic. Mutations in the PINK1 and PARKIN genes are responsible for juvenile-onset autosomal recessive PD variants of relatively mild progression [26]. Both factors are responsible for the autophagic degradation of damaged mitochondrial fragments [27]. Loss-of-function in this pathway leads not only to abnormal turnover of the mitochondria, but also affects the cellular susceptibility to invasion by bacterial pathogens such as *Mycobacterium tuberculosis* [28]. Mice with PINK1 or PARKIN mutations show consistent innate immune activation even in a special pathogen-free environment or upon cell culture, and the depletion of the cytosolic immune coordinator STING can prevent the neurodegenerative process in such mice [29, 30]. Thus, the investigation of ClpP-null triggered pathomechanisms may elucidate also frequent diseases.

An additional pathway on how mitochondrial dysfunction can modulate innate immunity was recently demonstrated in fumarate hydratase-deficient mice. Their cells exhibit excessively succinylated proteins in the mtDNA replication machinery, with progressive accumulation of misassembled nucleoids. Both fumarate and succinate are metabolites with a known role in the modulation of the cellular immune status [31, 32]. It is also known that several other factors, upon their release from bacteria/mitochondria, may be sensed by eukaryotic hosts as DAMPs and will trigger the innate immune responses [33]. Such factors include the metabolites ATP and cardiolipin, reactive oxygen species (ROS), *N-*formylated peptides, as well as enzymes-like cytochrome C and carbamoyl phosphate synthetase-1. It is unclear whether such mechanisms play a role in the immune activation of ClpP-null cells.

To govern mitochondrial endosymbiosis, nuclear transcription factors adapt intra-mitochondrial processes to cellular needs; therefore, sustained nuclear efforts will partially be useful to compensate mitochondrial anomalies. At the same time, however, they may modulate the expression of some cytosolic factors, and thus produce side effects throughout the cell over time. For example, transcriptional activity in the mitochondria is controlled (i) via autofeedback from mitochondrial TFAM (UniProt database entry P40630-1) to a nuclear isoform of TFAM (Uniprot entry P40630-2) [34, 35]. Additional regulation of mitochondrial transcription occurs via mitochondrial STAT1 that cross-talks with extra-mitochondrial STAT1; the latter will relocalize from the cytosol to the nucleus in a stimulus-dependent manner [36]. It is well established that nuclear STAT1 has a massive impact on the innate immune response.

Prior work from our labs demonstrated that mtDNA via the cGAS-STING pathway is primarily responsible for the heightened activation of type I interferon production and downstream upregulation of interferon-stimulated genes in cells and tissues from ClpP-null mice [9]. However, it is possible that other alterations (i.e., accumulation of unfolded proteins in the mitochondria with the release of *N*-formyl peptides, a profoundly altered metabolite profile, and/or extruded mitochondrial RNA with hypomethylation, nucleotide chains in imperfect supercoil structure) could further enhance innate immune activation in ClpP-null mice. This issue has major clinical consequences like the resistance of ClpP-null mice against ulcerative dermatitis with lethal outcomes [10].

Therefore, we examined the interplay of mitochondrial/nuclear protein isoforms, and we attempted to define the most affected DAMP-sensing pathways. Our data show strong accumulations for DNAJA3 (also known as TID1) and STAT1, intra-mitochondrially and extra-mitochondrially. Particularly, cytosolic nucleic acid sensors (higher number of RNA sensors than of DNA sensors) and several pattern recognition receptor families (number of elevated factors in RLR > TLR ≫ NLR) showed increased expression.

## Materials and methods

### Mouse breeding

Homozygous *ClpP*^−/−^ and wild-type (WT) mice were generated as littermate offspring from heterozygous breeders. They were genotyped at postnatal day 10 by ear-punches. Immediately after weaning, pairs of mutants with age-/sex-matched WT controls were housed together, aged, and dissected, as reported before [10]. The mice used were kept under FELASA-certified conditions at the Central Animal Facility (ZFE) of the Goethe University Medical Faculty in Frankfurt. All animal experiments were carried out in accordance with the German Animal Welfare Act and with the approval of the local animal authorities (Regierungspräsidium Darmstadt, FK/1073).

### Mouse embryonic fibroblast generation and culture

Generation and culture of mouse embryonic fibroblasts (MEFs) were done as previously described [11]. Following intercrosses of *ClpP*^+/−^ mice, WT and *ClpP*^−/−^ MEFs were prepared from individual embryos at 14.5 days post-coitus. Cells were cultivated in Dulbecco’s modified Eagle’s medium (DMEM) (Gibco, Thermo Fisher Scientific, Waltham, MA, USA) supplemented with 15% fetal bovine serum (FBS) (Gibco, Thermo Fisher Scientific) and 1% l-glutamine (Gibco, Thermo Fisher Scientific) at 37 °C and 5% CO_2_ in a humidified incubator.

### Mouse brain native gel electrophoresis and complexome profiling

Sample preparation [37] and high-resolution native electrophoresis (hrCNE) [38] of brain tissue were essentially done as described. Briefly, the brains were taken and further disrupted using a pre-cooled motor-driven glass/Teflon Potter-Elvehjem homogenizer at 2000 rpm and 40 strokes. Homogenates were centrifuged for 15 min at 600*g* to remove the nuclei, cell debris, and intact cells. The mitochondrial membranes were sedimented by centrifugation of the supernatant for 15 min at 22,000*g*. The mitochondria-enriched membranes from 10 mg brain tissue were resuspended in 35 μl solubilization buffer (50 mM imidazole pH 7, 50 mM NaCl, 1 mM EDTA, 2 mM aminocaproic acid) and solubilized with 20 μl 20% digitonin (Serva, Heidelberg, Germany). Samples were supplemented with 5 μl 0.1% Ponceau S in 50% glycerol. Equal protein amounts of samples were loaded on top of a 3 to 18% acrylamide gradient gel (dimension 14 × 14 cm). After native electrophoresis in a cold chamber, native gels were fixed in 50% (v/v) methanol, 10% (v/v) acetic acid, and 10 mM ammonium acetate for 30 min and stained with Coomassie (0.025% Serva Blue G, 10% (v/v) acetic acid) or blotted onto the PVDF membranes and used for antibody decoration using an antibody against NDUFB8 and MitoProfile Total OXPHOS Rodent WB Antibody Cocktail (Mitosciences, Eugene, OR, USA). Coomassie-stained lanes were fractionated in 60 even pieces and digested with trypsin and subsequently analyzed by mass spectrometry. Experimental details and data were deposited to dataset identifier PRIDE: PXD025478.

### Reverse transcriptase real-time quantitative PCR

As described in previous studies [10] and following the manufacturers’ instructions, total ribonucleic acid (RNA) isolation from MEFs and brain tissues was performed with TRI reagent (Sigma-Aldrich, St. Louis, MO, USA), and reverse transcription by SuperScript IV VILO Master Mix (Thermo Fisher Scientific). Reverse-transcriptase real-time quantitative polymerase chain reaction (RT-qPCR) was carried out with TaqMan® Gene Expression Assays (Thermo Fisher Scientific) in complementary deoxyribonucleic acid (cDNA) from 10 ng total RNA in 10 μl reactions with 2× Master Mix (Roche, Basel, Switzerland, and Thermo Fisher Scientific) in a StepOnePlus Real-Time PCR System (Applied Biosystems, Thermo Fisher Scientific). The data was analyzed with the 2^−ΔΔCT^ method [39]. To verify the null mutation in used tissue and MEF samples, RT-qPCR assays of *ClpP* normalized to *Tbp* (Tata-box binding protein) were performed, in addition to the genotyping of each animal. The following TaqMan assays (Thermo Fisher Scientific) were employed to quantify the individual messenger RNA (mRNA) levels: *Aim2*- Mm01295719_m1, *ClpP*- Mm00489940_m1, *Ddx58-*Mm01216853_m1, *Dnaja3*- Mm00469723_m1, *Eif2ak2-*Mm01235643_m1, *Gbp3*- Mm00497606_m1, *Ifi204-*Mm00492602_m1, *Ifi205b (=Mnda)*- Mm04204353_mH, *Ifi35*- Mm00510329_m1, *Ifi44*- Mm00505670_m1, *Ifih1-*Mm00459183_m1, *Ifit1* (= *Isg56*)- Mm00515153_m1, *Ifit3-*Mm01704846_s1, *Ifna1*- Mm03030145_gH, *Ifnb1-*Mm00439552_s1, *Irf3*- Mm00516784_m1, *Mavs* (=*Ips-1*)-Mm00523170_m1, *Mb21d1 (=cGas)*- Mm01147496_m1, *Nfkb1*- Mm00476361_m1, *Nlrp3*- m00840904_m1, *Nlrx1-*Mm00617978_m1, *Oas1b*- Mm00449297_m1, *Oasl2-*Mm00496187_m1, *Rsad2*- Mm00491265_m1, *Stat1-*Mm00439531_m1, *Stat2*- Mm00490880_m1, *Tbp-*Mm00446973_m1, *Tlr3*- Mm01207404_m1, *Tlr9-*Mm00446193_m1, *Tmem173*- Mm01158117_m1, *Trim25-*Mm01304226_m1, *Trim30a*- Mm00493346_m1, *Trim56-*Mm01207494_m1, and *Tspan6*- Mm00451045_m1.

### Quantitative immunoblotting

Protein extraction and sample preparation from brain tissues and MEFs were carried out as described before [10, 40]. Samples of 20 μg of total protein were mixed with 2× loading buffer [250 mM Tris/HCl (pH 6.9), 20% glycerol, 4% SDS (sodium dodecyl sulfate), 10% β-mercaptoethanol, 0.01% bromophenol blue, and 5% MilliQ water]; heated at 90 °C for 5 min; and afterward separated in 8%, 10%, 12%, or 15% tris-glycine polyacrylamide gels depending on the size of the investigated protein. Precision Plus Protein^™^ All Blue Standards (Bio-Rad, Hercules, CA, USA) was used as a size marker. Following the gel electrophoresis, protein transfer to nitrocellulose membranes (Bio-Rad) was done at 50 V over 90 min. The membranes were blocked in 5% BSA/TBS-T (Tris-buffered saline/Tween 20) for 1 h at room temperature (RT) and incubated over night at 4 °C with primary antibodies against ClpP (1:1000, Proteintech, Rosemont, IL, USA, 15698-1-AP), DDX58 (1:1000, Cell Signaling, Danvers, MA, USA, #3743S), DNAJA3 (1:500, Cell Signaling, #4775), IFIT3 (1:800/1:1000, Proteintech, 15201-1-AP), IκBα (1:1000, Cell Signaling, #4814), IKKα (1:500, Cell Signaling, #11930), IKKβ (1:1000, Cell Signaling, #8943), IRF3 (1:1000, Cell Signaling, #4302), IRF7 (1:1000, Abcam, Cambridge, UK, ab109255), ISG15 (1:1000, Invitrogen, PA5-17461), NFκB P65 (1:1000, Cell Signaling, #8242), Phospho-IKKα/β (1:800, Cell Signaling, #2697), Phospho-IκBα (1:1000, Cell Signaling, #2859), STAT1 (1:1000, Cell Signaling, #9172), TLR9 (1:800, Novus B, Centennial, CO, USA, NBP2-24729), and TRIM25 (1:1000, Abcam, ab167154). As secondary antibodies, fluorescence-labeled anti-rabbit or anti-mouse antibodies (1:15,000, Thermo Fisher Scientific, Invitrogen) were used. The fluorescence was detected by using the Li-Cor Odyssey Classic Instrument and was densitometrically analyzed with Image Studio Lite version 5.2 (Li-Cor Biosciences). Bands were normalized against β-ACTIN (= ACTB (β-actin)) (1:10,000, Sigma-Aldrich, A5441). For fractionation experiments, GAPDH (1:10,000, Sigma-Aldrich, Taufkirchen, Germany, #CB1001), LAMIN A/C (1:1000, Abcam, #AB169532), or PORIN-1 (1:500, Cell Signaling, #4866) were used as loading controls.

### Subcellular fractionation

Fractionation into nuclear, mitochondrial, and cytosolic fractions was done as follows: 4 × 10^6^ MEF cells were collected with trypsin, centrifuged at 800 *g* for 3 min, and washed with PBS; the centrifugation step was repeated; and the pellet was resuspended in 300 μl cytosol extract buffer (CEB). The suspension was shaken 5 min head-to-head at RT and centrifuged at 800 *g* for 3 min. The supernatant was stored as cytosolic fraction. The remaining pellet was washed once with CEB, centrifuged at 800 *g* for 3 min and then resuspended in 300 μl mitochondrial lysis buffer (MLB), shaken 10 min head-to-head at RT, and centrifuged at 800 *g* for 3 min. The supernatant was stored as a mitochondrial fraction. The remaining pellet was washed once with MLB, centrifuged at 800 *g* for 3 min and then resuspended in 300 μl RIPA buffer. The suspension was sonicated, 10 min shaken head-to-head at RT, and centrifuged at 800 *g* for 3 min. The supernatant was stored as a nuclear fraction. Experiments were repeated with 3 WT and 3 *ClpP*^−/−^ lines. Buffers were composed as follows:

CEB: 250 mM sucrose, 70 mM KCl, 137 mM NaCl, 4.3 mM Na2HPO4, 1.4 mM KH2PO4, with freshly added 100 μM PMSF, 10 μg/ml leupeptin, 2 μg/ml aprotinin, 200 μg/ml digitonin
MLB: 50 mM TRIS/HCl pH 7.4, 150 mM NaCl, 2 mM EDTA, 2 mM EGTA, 0.2% Triton X100, 0.3% NP40, with freshly added 100 μM PMSF, 10 μg/ml leupeptin, 2 μg/ml aprotinin
RIPA buffer: 50 M TRIS/HCl pH 8.0, 150 mM NaCl, 0.1% SDS, 1% triton, 0.5% sodium deoxycholate, 2 mM EDTA, protease inhibitor cocktail (Sigma Aldrich, St. Louis, MO, USA).

All chemicals were purchased from Merck (Darmstadt, Germany) unless mentioned otherwise.

### Bioinformatic and statistical evaluation

For the bioinformatic assessment of protein associations and the enrichment within specific pathways, the BioGrid database (https://thebiogrid.org/, accessed on June 8, 2021) list of known interactors of human DNAJA3 was evaluated by the webtool STRING (https://string-db.org/, accessed on June 8, 2021).

Statistical analysis of quantitative immunoblot and RT-qPCR results were conducted by using GraphPad Prism (version 8.4.2, GraphPad, San Diego, CA, USA) with unpaired Student’s *t*-tests. The results including standard error of the mean (SEM) and *p* values (*p*= probability) were visualized in bar graphs, with the following significances illustrated by asterisks or symbols: * (or #/§) *p* < 0.05; ** (or ##/§§) *p* < 0.01; *** (or ###/§§§) *p* < 0.001; **** (or ####/§§§§) *p* < 0.0001; not significant (ns)) *p* > 0.05; tendency (T) 0.05 < *p* < 0.1

### Visualization

Graphs were generated by using GraphPad Prism (version 8.4.2). Figures were created using Microsoft Powerpoint (Version 2016) and Adobe Photoshop (Version CS2).

## Results

### Protein mass spectrometry analysis of mitochondrial matrix unfolded protein response factors by complexomics reveals prominent assembly anomalies for DNAJA3 in ClpP^−/−^ mouse brain

**Fig. 2:**
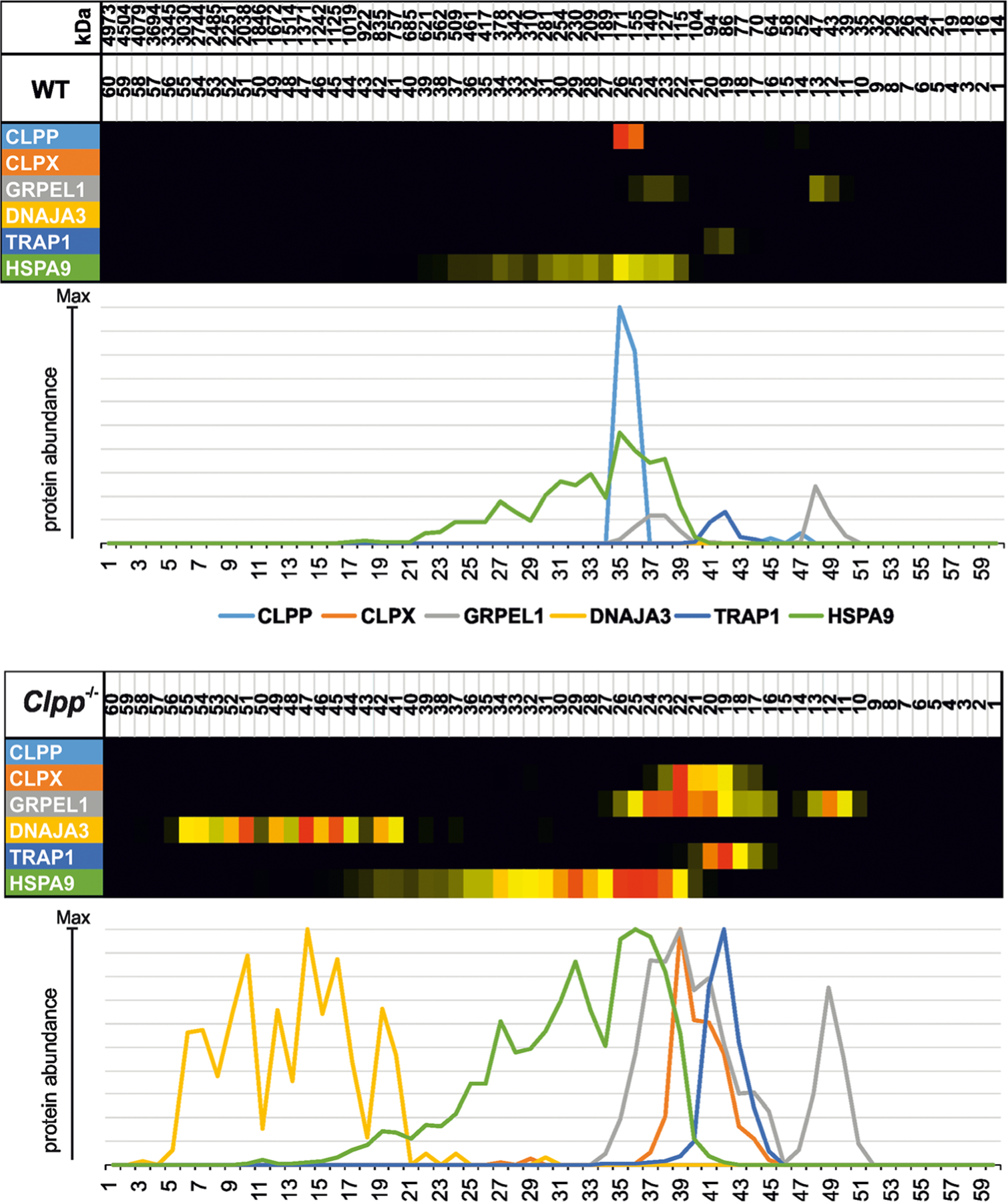
Complexome profile of mitochondrial matrix chaperones in ClpP^−/−^ brain. Digitoninsolubilized mitochondrial complexes were separated by high-resolution clear native electrophoresis (hrCNE) into 60 fractions and identified/quantified by mass spectrometry. Protein complexes in Coomassie-stained gels were further processed to present abundance profiles as a heatmap (with red > yellow color representing the highest abundance) and 2D plot (with each protein represented by differently colored line as coded in gel picture above). The absence of ClpP is clearly detected, and triggers not only marked accumulation of several downstream factors in the UPR^mt^ pathway, but also abnormal migration at unexpected high molecular sizes, particularly for the strongly accumulated co-chaperone DNAJA3

To understand the cumulative effects of chronic ClpP deficiency on the UPR^mt^ pathway in brain tissue with its postmitotic neurons during the aging process, brains from 12-month-old *ClpP*^−/−^ and sex-matched littermate WT mice were dissected. The tissue was homogenized and centrifuged to obtain mitochondria-enriched membranes. High-resolution native electrophoresis resolved protein complex assemblies, and subsequent mass spectrometry identified and quantified their components (Figure 2). The selective analysis of UPR^mt^ factors confirmed the absence of ClpP (monomer with size 29 kDa); furthermore, it detected pronounced accumulations for the disaggregase ClpX (69 kDa) and the Hsp90-homologous molecular chaperone TRAP1 (size 80 kDa). Interestingly, an accumulation with additional disperse migration at abnormally high molecular weights was evident for several UPR^mt^ proteins: the Hsp70-homologous chaperone HSPA9 (Mortalin, 73 kDa), its GrpE-homologous co-chaperone GRPEL1 (24 kDa), and even more so for its DnaJ-homologous co-chaperone DNAJA3 (52 kDa for large isoform precursor, 49 kDa for small isoform precursors).

DNAJA3 was previously shown to be co-regulated with ClpP in the mitochondrial unfolded protein response pathway. It might be a degradation substrate of ClpP or might co-accumulate with such substrates in the mitochondrial matrix. DnaJ was originally identified in *E. coli* bacteria as a factor required for the replication of bacterial and viral DNA [41], so this protein family has the potential to influence nuclear events.

### Known protein interactions show DNAJA3 enriched in mitochondria, but also in pathways of the nucleus, host-virus interaction, and spinocerebellar ataxia

To survey the protein complexes that might be preferentially impacted by excess DNAJA3, we surveyed available knowledge in the BioGrid database, where the associations of endogenous and overexpressed DNAJA3 in various cell types are listed. Statistical evaluation by the STRING webtool detected a highly significant enrichment of diverse pathways and compartments (Suppl. Table S1). Among PFAM interaction motifs, the HSP70 family stood out in confirmation of the DNAJA3 role as co-chaperone. Prominent enrichments with high significance included mitochondrial translation among GO terms “cellular process,” respiratory NADH dehydrogenase (ubiquinone) activity among GO terms “function,” and mitochondrial nucleoid among GO terms “cell component”, in excellent agreement with the observation of altered turnover of these assemblies upon ClpP mutation [8, 9, 42]. Interestingly, the nucleus and nucleolus factors were also significant. Among “keywords,” host-virus interaction factors were enriched, as well as spinocerebellar ataxia proteins. Also, Leigh syndrome; Parkinson’s, Huntington’s, and Alzheimer’s diseases; and neurodegeneration factors in general were enriched with significance among “keywords” or “KEGG” pathways. The interaction network of DNAJA3 as illustrated by the STRING algorithm is shown in Suppl. Fig. S2, where its association with the ClpXP complex appears in the lower left, while its association with inflammatory factors such as JAK2, IFNGR2, IRAK1, NFKBIB, NFKBIA, and other host-virus interaction pathway components appears in the upper left.

DNAJA3 was observed in different experimental approaches to modulate JAK-STAT signals and to mediate strong transcriptional induction of specific interferon-stimulated genes [23, 43, 44], possibly via direct protein interaction [45, 46]. The modulation of inflammatory responses not only via STAT1 and STAT3, but also via NFκB have already been validated in functional studies [47–49]. Overall, the data confirm that DNAJA3 accumulation might be a relevant mediator of inflammatory responses in ClpP-null cells. Furthermore, the novel enrichment of neurodegeneration networks among DNAJA3 is compatible with the idea that the ataxia and neuropathy of ClpP-mutant patients are modulated by DNAJA3 accumulation.

Given that any tissue inflammation might be caused by the innate immune system or the adaptive immune system in the bloodstream, we decided to perform further studies in primary fibroblast cultures taken at early embryonic age from *ClpP*^−/−^ mice and their sex-matched WT littermates. These results were compared to data from brain samples at two ages.

### Validation of ClpP and DNAJA3 protein/mRNA level change in MEFs versus the brain

**Fig. 3:**
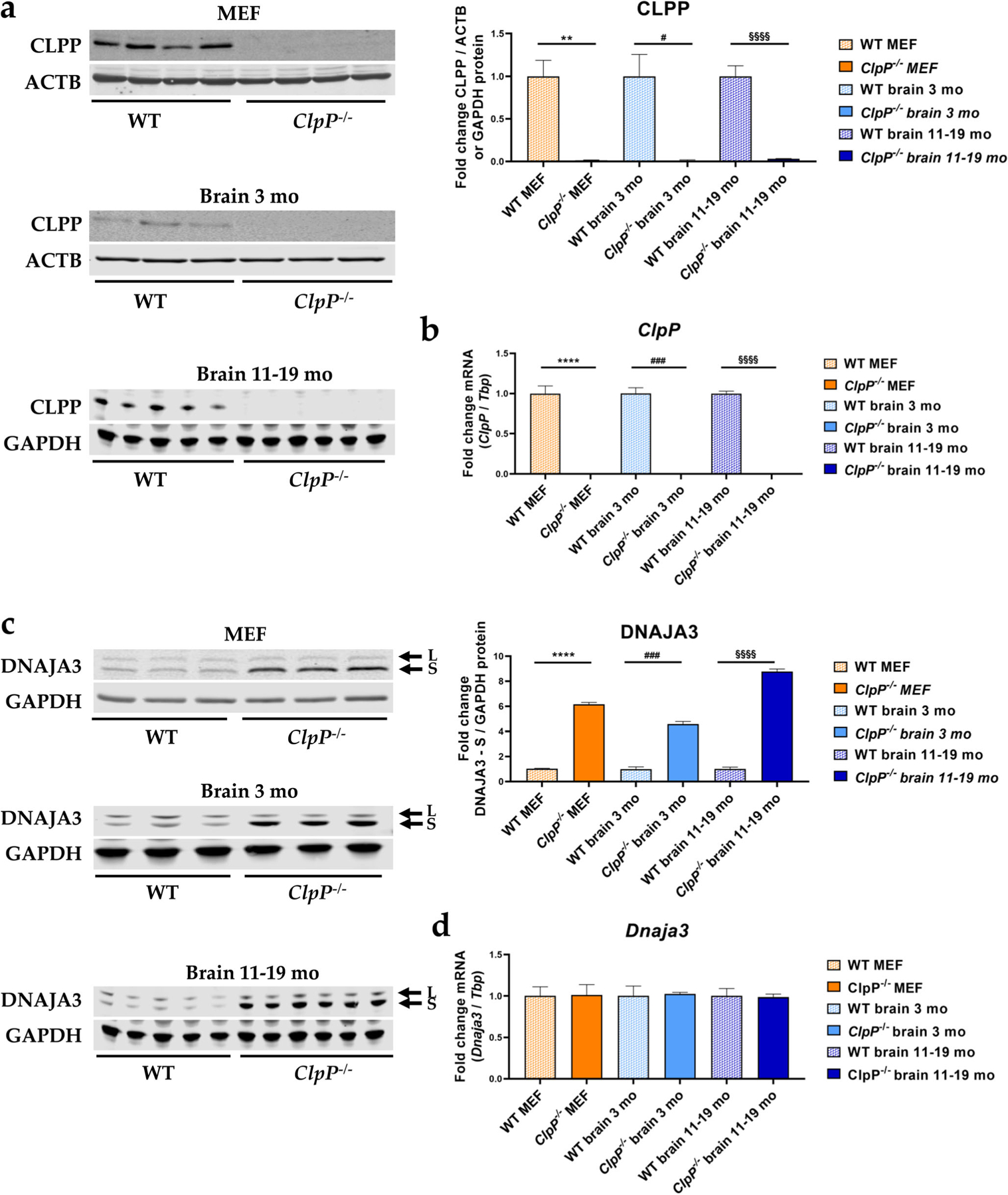
Verification of *ClpP*^−/−^ genotype via **a** quantitative immunoblots and **b** RT-qPCRs and analysis of **c** DNAJA3 protein and **d** *Dnaja3* mRNA levels, in MEFs and brain tissue of 3 versus 11–19-month-old mice. Immunoblots were normalized to ACTB or GAPDH, RT-qPCRs to *Tbp* levels. Data are presented as mean ± SEM. WTs are shown as checked, and ClpP^−/−^ as plain colored bar graphs. Significances are illustrated by symbols: ** or §§ *p* < 0.01; ### *p* < 0.001; **** or §§§§ *p* < 0.0001; not significant (ns) *p* > 0.05. Asterisks portray significant differences between WT and ClpP^−/−^ MEF, hashtags show significant effects in 3-month-old brain tissue between WT and *ClpP*^−/−^ mice, and section signs visualize significant differences in brain tissue from 11–19-month-old mice, between WT and *ClpP*^−/−^ genotypes. WT MEF: *n*= 4–5; *ClpP*^−/−^ MEF: *n*= 3–5; WT brain 3 months: *n*= 3; *ClpP*^−/−^ brain 3 months: *n*= 3; WT brain 11–19 months: *n*= 5; *ClpP*^−/−^ brain 11–19 months: *n*= 6

First, the effects of genetic *ClpP*-ablation were assessed at mRNA and protein level by immunoblots and RT-qPCRs, confirming the ClpP deficiency in MEFs and the brain samples used (Figure 3a, b).

With quantitative immunoblots, a strong accumulation was again found for DNAJA3 (Figure 3c). This observation was not explained by transcriptional induction (Figure 3d), so it might simply represent a marker of protein complex assembly problems that result in slowed turnover and degradation of DNAJA3 and its targets.

### Cell fractionation demonstrates ClpP deficiency to increase cytosolic STAT1 and nuclear DNAJA3

**Fig. 4:**
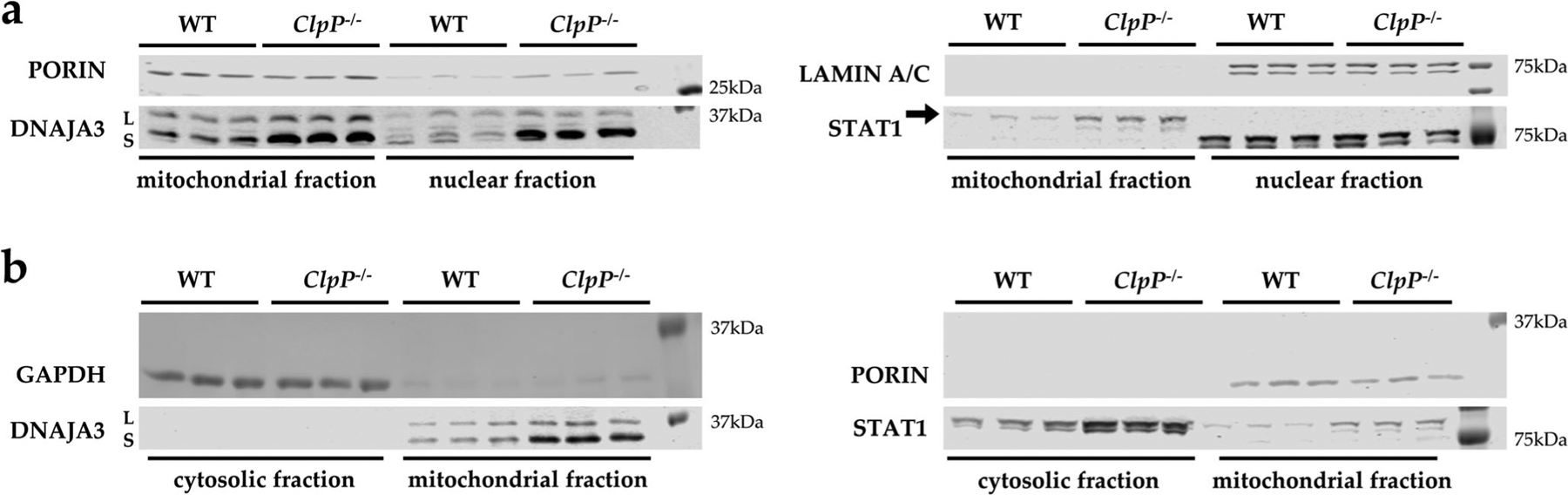
Cell fractionation in MEFs, comparing mitochondrial with nuclear (**a**) and cytosolic (**b**) compartments. Immunoblotting was used to control the purity of the fractionation with the mitochondrial marker PORIN, the nuclear marker LAMIN A/C, and the cytosolic marker GAPDH, comparing littermate WT and *ClpP*^−/−^ cells of matched sex. The detection of DNAJA3 revealed ClpP deficiency to cause accumulation of DNAJA3 short isoform (S) in the mitochondria and nucleus, while STAT1 (87 kDa) accumulation occurred in the mitochondria and cytosol

It is unclear how ClpP deficiency and UPR^mt^ pathology signal in a retrograde manner to dysregulate factors with nuclear function, such as the transcription factor STAT1, already at the embryonal stage in fibroblasts. To elucidate this issue, the fractionation of different subcellular compartments by sequential protein extraction with appropriate detergents was done. The enrichment of the mitochondrial fraction was assessed by PORIN as a component of the main outer mitochondrial membrane multimeric pore; the cytosolic fraction was assessed by GAPDH as a glycolytic enzyme, and the nuclear fraction by LAMIN A/C as a component of the matrix on the inner surface of the nuclear envelope. Immunoblot detection with DNAJA3 antibodies showed an accumulation for the mitochondrially imported and cleaved small isoform not only in the *ClpP*^−/−^ mitochondrial fraction; also, in the *ClpP*^−/−^ nuclear fraction a band of very similar size was accumulated (Figure 4a). The DNAJA3 small isoform in the *ClpP*^−/−^ mitochondrial fraction showed its elevated abundance also on the membranes where more protein was loaded per lane, in comparison with the cytosolic fraction (Figure 4b) where DNAJA3 was below the detection threshold.

In contrast, STAT1 immunoproduct of the predicted size accumulated in *ClpP*^−/−^ mitochondrial fraction and in *ClpP*^−/−^ cytosolic fraction (Figure 4b). In the nuclear fraction, STAT1 was below the detection threshold, possibly due to the insufficient solubilization from DNA complexes (Figure 4a).

It is known that ClpP-null cells have elevated levels of misassembled mtDNA [9, 10], so it seems appropriate to observe responses of a mammalian homolog of DnaJ chaperones which are known to control bacterial and viral DNA replication [41, 50]. STAT1 was reported to repress mitochondrially encoded transcripts [36], so its increased abundance may be a direct effort to compensate for the elevated mtDNA dosage and transcription in ClpP-null cells; alternatively, it might be a downstream effect of its interactions with DNAJA3. Importantly, these responses involve not only intra-mitochondrial DNAJA3 and STAT1 but extend to extra-mitochondrial compartments.

### Transcriptional analysis of induced ISGs, PRRs, and associated factors

**Fig. 5:**
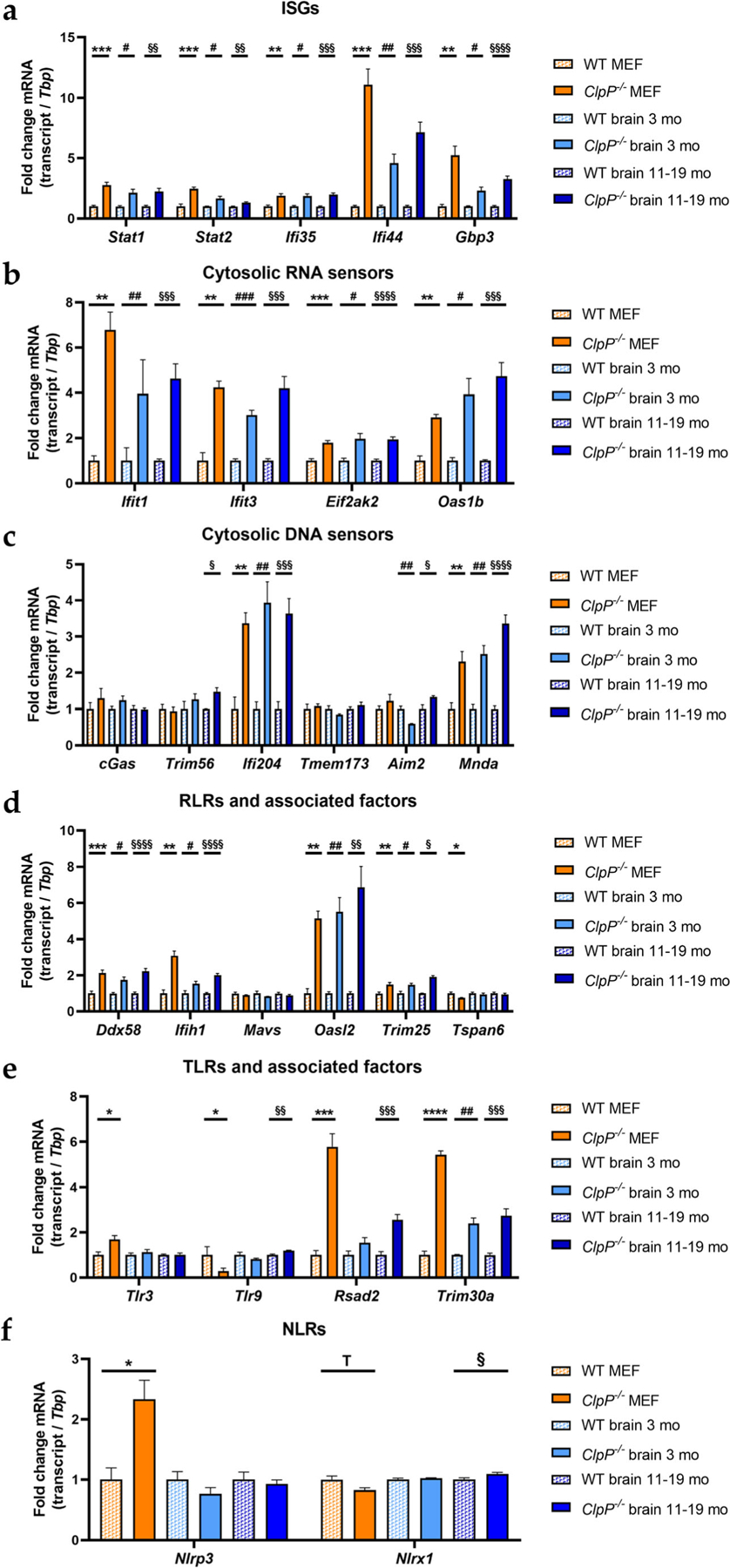
Analysis of mRNA expression by RT-qPCR in MEFs and brain tissue of 3- and 11–19-month-old mice, focusing on **a** induced interferon-stimulated genes, **b** cytosolic RNA sensors, **c** cytosolic DNA sensors, **d** RLRs with associated factors, **e** TLRs with associated factors, and **f** NLRs. RT-qPCRs were normalized to *Tbp* levels. Data are presented as mean ± SEM. WTs are shown as checked, and *ClpP*^−/−^ as plain colored bar graphs. Statistical tendencies and significances are illustrated by symbols: * or #/§ *p* < 0.05; ** or ##/§§ *p* < 0.01; *** or ###/§§§ *p* < 0.001; **** or §§§§ *p* < 0.0001; not significant (ns) *p* > 0.05; tendency (T) 0.05 < *p* < 0.1. Asterisks portray significant differences between WT and *ClpP*^−/−^ MEF, hashtags illustrate significant effects in brain tissue between 3-month-old WT and *ClpP*^−/−^ mice, and section signs visualize significant differences in brain of 11–19-month-old WT versus *ClpP*^−/−^ mice. WT MEF: *n*= 3–9; *ClpP*^−/−^ MEF: *n*= 3–8; WT brain 3 months: *n*= 3; *ClpP*^−/−^ brain 3 months: *n*= 3; WT brain 11–19 months: *n*= 5; *ClpP*^−/−^ brain 11–19 months: *n*= 5–6

In view of published global transcriptome evidence of increased antiviral defenses in ClpP-null mice, we wanted to further characterize the signaling pathways involved [9–11]. Therefore, mRNA expression levels of relevant transcription factors, interferon-stimulated genes (ISGs), cytosolic DAMP sensors, and pattern recognition receptors (PRRs) with associated factors were surveyed by RT-qPCR (Figure 5). These factors were grouped in alternative pathways, taking into account their reclassification by recent literature [51–54].

Several investigated inflammatory transcription factors and ISGs (*Stat1*, *Stat2*, *Ifi35*, *Ifi44*, *Gbp3*) showed significantly increased mRNA levels (Figure 5a). Very strong inductions were detected for *Ifi44* (effect sizes of 7.257-fold in the brain of 11–19-month-old ClpP-null mice versus 11.110-fold in MEFs). The PRRs lead to an induction of ISGs, via type I interferons (IFN-I) and other pathways. Some of the ISGs are the PPRs themselves, which may lead to an effect of self-induction. Other ISGs also exert anti-pathogenic functions in the context of the innate immune system. The main IFN-I transcripts, *Ifna1* and *Ifnb1*, showed only partially significant inductions (Figure S2a). While both mRNAs were significantly upregulated in MEFs, only *Ifnb1* was induced in the 11–19-month-old ClpP-null mouse brain tissue samples.

Prominent significant upregulations were also found for several cytosolic nucleic acid sensors (*Ifit1*, *Ifit3*, *Oas1b*, *Ifi204*, *Mnda*, with lesser induction for *Eif2ak2=Pkr* and for *Aim2=Pyhin4*) (Figure 5b, c), and the retinoic acid-inducible gene like receptors (RLRs) (*Ddx58=Rig-I*, *Ifih1*) with their associated factors (*Oasl2*, *Trim25*) (Figure 5d). No significant transcriptional changes were apparent in the cGAS-STING signaling pathway, with STING mRNA here referred to as *Tmem173*, consistent with previous observations that the regulation of this pathway occurs via dimerization and phosphorylation (Figure 5c). Nonetheless, *Trim56* mRNA levels showed a 1.473-fold induction in 11–19-month-old ClpP-null mice. TRIM56 protein can induce cGAS and therefore the downstream signaling pathway [55]. Although a 2.261-fold induction of *Ddx58* and a 2.016-fold induction of *Ifih1* were detected in the brain tissue of 11–19-month-old *ClpP*^−/−^ mice (Figure 5d), the downstream mitochondrial adapter *Mavs* was not significantly altered in the expression. Interestingly, toll-like receptors (TLRs) showed fewer transcriptional inductions (Figure 5e), and nuclear oligomerization domain-like receptors (NLRs) almost none (Figure 5f). In young brains, only *Trim30a* levels were significantly increased (Figure 5e). In contrast, the interferons *Ifna1* and *Ifnb1*, as well as *Nfkb1* and *Irf3*, did not show transcriptional activation (Suppl. Figure S2a–c).

### Quantitative immunoblots of induced transcription factors, ISGs, nucleic acid sensors, and PRRs

**Fig. 6:**
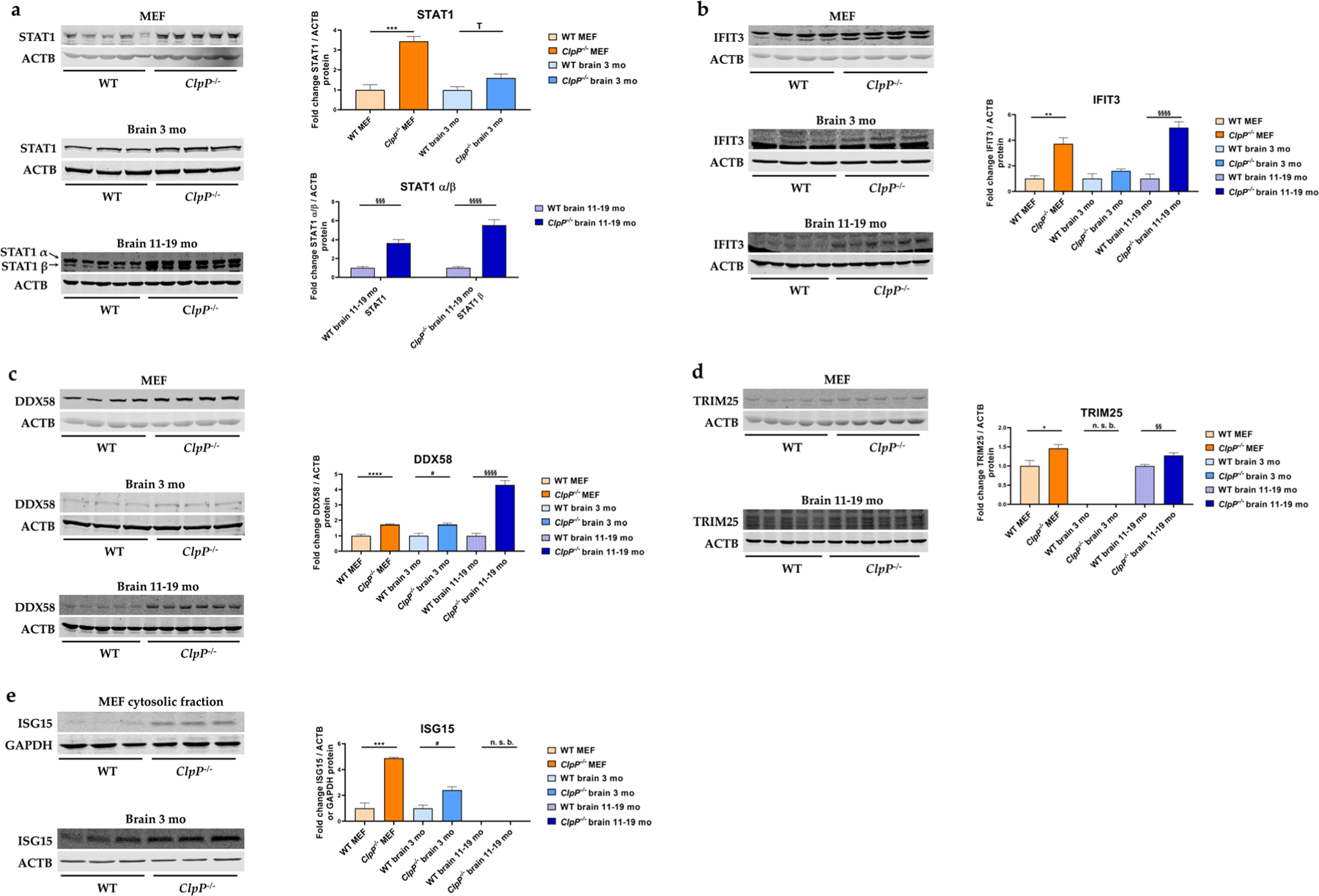
Analysis of protein expression by quantitative immunoblots for **a** the transcriptional immune modulator STAT1, **b** the cytosolic RNA sensor IFIT3, as well as the RLR pathway components **c** DDX58, **d** TRIM25, and **e** ISG15, in MEFs and brain from 3- versus 11–19-month-old mice. Immunoblots were normalized to ACTB or GAPDH. Data are presented as mean ± SEM. In bar graphs, WT is shown as checked, while *ClpP*^−/−^ as plain colored. Statistical significances are illustrated by symbols: * or # *p* < 0.05; ** or §§ *p* < 0.01; *** or §§§ *p* < 0.001; **** or §§§§ *p* < 0.0001; not significant (ns) *p* > 0.05. Asterisks portray significant differences between WT and *ClpP*^−/−^ MEFs, hashtags show significant effects in the brain between 3-month-old WT and *ClpP*^−/−^ mice, and the section sign visualizes significant differences in the brain from 11–19-month-old WT versus *ClpP*^−/−^ mice. n. s. b., no specific bands. WT MEF: *n*= 3–5; *ClpP*^−/−^ MEF: *n*= 3–5; WT brain 3 months: *n*= 3; *ClpP*^−/−^ brain 3 months: *n*= 3; WT brain 11–19 months: *n*= 5; *ClpP*^−/−^ brain 11–19 months: *n*= 6

To validate the significant and strong transcript dysregulation at the protein level, quantitative immunoblots were employed whenever sufficiently specific and sensitive antibodies were commercially available. The significantly elevated abundances of STAT1, IFIT3, DDX58, TRIM25, and ISG15 in ClpP-null MEFs confirmed the activation of the innate immune defenses from the embryonal stage, independent from the adaptive immune system (Figure 6). The significantly elevated abundances of STAT1, DDX58, and ISG15 in the brains of 3-month-old ClpP-null mice demonstrated this inflammation in nervous tissue to precede neural phenotypes; in the mouse model of Perrault syndrome, the neurodegeneration features such as hearing loss, ataxia, and white matter degeneration do not appear before ages around 12 months [10]. The even stronger increases of STAT1, IFIT3, and DDX58 abundances in the brains of 11–19-month-old ClpP-null mice indicate that their levels correlate with the progression of sterile inflammation in the nervous tissue. Other factors in the innate immune sensing pathways, such as the interferons IRF3 and IRF7; several NFκB-associated factors; and TLR9 did not exhibit elevated abundance (Suppl. Figure S2c–e).

## Discussion

**Fig. 7:**
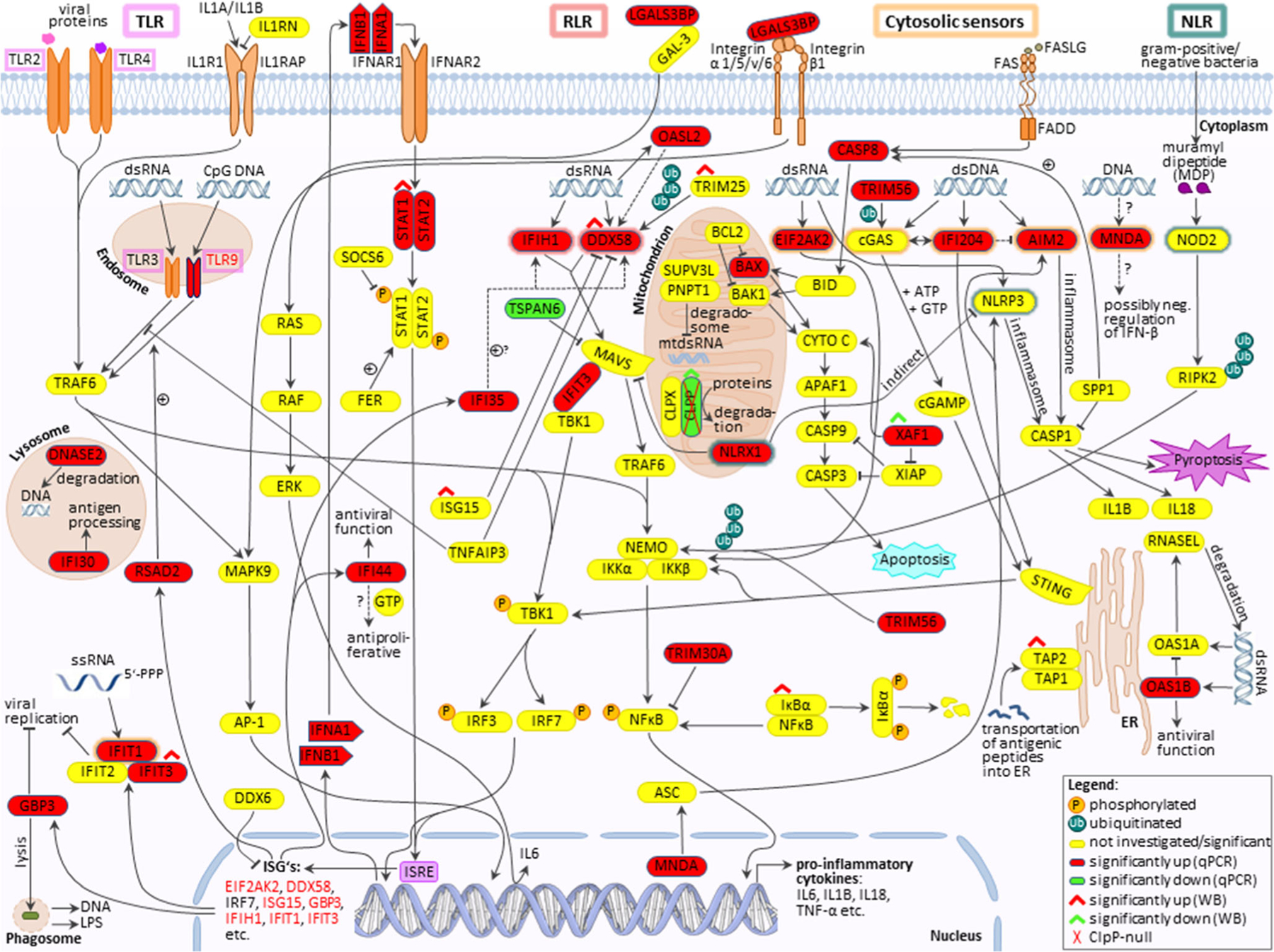
Schematic overview of innate immunity signaling, with an illustration of the results from this study. TLRs= outlined in pink; RLRs= outlined in red; cytosolic nucleic acid sensors= outlined in orange; NLRs= outlined in green. Significant RT-qPCR results are represented by highlighting the factors in red, green, or yellow (red= induced; green= reduced; yellow= not significant/investigated). Significantly altered protein expressions were marked with red or green roofs (red= increased; green= reduced). Dashed lines indicate discussed functions of corresponding factors. +, induction; P, phosphorylated; Ub, ubiquitinated; other symbols can be retrieved in the GeneCards database

We attempted to elucidate the molecular pathways how the deficiency of ClpP as a key molecule in the UPR^mt^ triggers innate immune activation. The novel data obtained identify DNAJA3 and STAT1 accumulations intra- and extra-mitochondrially as possible crucial mediators; they also reflect a quite specific inflammatory profile rather than a generic induction of interferon-stimulated genes. The latter is readily apparent by the comparison of the 2-fold induction of *Ifi35* mRNA versus the >10-fold induction of *Ifi44* mRNA (Figure 5a) or the comparison of the strong induction of many RLR factors with up to 7-fold effect size (Figure 5d) versus the singular induction of an NLR factor in MEFs (Figure 5f). This profile seems tuned to optimally detect immune-stimulatory dsDNA and dsRNA, by transcriptional activation of cytosolic DNA and RNA sensors, RLR pathway components, and ISGs. A detectable sensitization is also apparent towards ssRNA, while the sensing factors for muramyl dipeptides from Gram-negative bacteria appeared unchanged (Figure 7). This can be explained by the previously reported accumulation of mitoribosomes and mitochondrial nucleoids in ClpP-null cells [8, 9], which may act as damage-associated molecular patterns. We have previously shown by cGAS- and STING- depletion of ClpP-null cells that the ISG induction mostly depends on the mtDNA [9], believing that the mtRNA extrusion to the cytosol would make only a minor contribution. The novel observation that many RNA sensors are also activated might simply reflect a general antiviral program, given the large number of very diverse RNA viruses. However, our data can be viewed also in light of a recent publication that demonstrated cGAS/STING to be co-activated by collided ribosomes and translation stress [56]. How could such translation problems inside the mitochondria be sensed outside and trigger nuclear responses? Technically, it is very difficult to detect mtDNA or mtRNA extrusion to the cytosol without the possibility of artifacts. It is conceivable that the enlarged nucleoids and stalled mitoribosomes in ClpP-null cells do not lead to *bona fide* release of mtDNA and mtRNA. Instead, they might result in inner membrane herniation as has been reported before [57], with cGAS/STING sensing around the periphery of mitochondria. Clearly, the inflammatory response in ClpP-null cells depends on the mitochondrial VDAC pores, as shown with VBIT-4 inhibitor experiments in our previous study [9]. Such a mitochondria-triggered inflammation scenario is known from TFAM heterozygous knockout cells, appears similarly strong in ClpP homozygous knockout cells, and appears considerably weaker in PINK1/PARKIN mutants, when the expression upregulation levels and the delay in pathology onset are compared.

Previously, the concept prevailed that the Perrault syndrome’s late manifestations of neurodegeneration and deafness are explained by the impaired translation fidelity in the mitochondria, which leads to bioenergetics deficits [8, 58]. If mitochondrial mishandling of nucleic acid/protein complexes triggers innate immune activation and if the inflammatory process in the nervous tissue is responsible for deafness, ataxia, and leukodystrophy in the later course of Perrault syndrome, then the therapeutic injection of antisense oligonucleotides to deplete STING as an integrator of inflammatory signaling might have neuroprotective value. Preventive benefits of STING depletion have already been demonstrated in PINK1 and PARKIN mutants where the neurodegenerative process could be mitigated [30]. The therapeutic efficacy of antisense oligonucleotide (ASO) injections is impressive in patients and animal models with motor neuron degeneration, if the administration can be done at the earliest ages before the atrophy occurs [59, 60].

The mechanisms of innate immunity activation by toxic nucleic acids appear crucial for a host of neurological disorders. These include mutations in TWNK, POLG, TFB1M, MTRNR1, TRMU, and GTPBP3 or in the components of the mitochondrial and nuclear DNA repair pathway such as RRM2B, or in the cytosolic ribosomal translation machinery amino acid tRNA synthetases. This notion is consistent with the existence of autoimmune disorders where the tRNA synthetases are targeted specifically by autoantibodies, resulting in early-onset myopathy, interstitial lung inflammation, skin rash, arthropathy, and vasculitis [61–65]. Certainly in the autoimmune vasculitis variants known as Aicardi-Goutières syndrome, where altered degradation of cytosolic DNA or RNA is caused by mutations in TREX1/SAMHD1/RNASEH2A/B/C/ADAR/IFIH1/PNPT1, the progressive immune activation leads to phenotypes of neuro-inflammation/-degeneration [66, 67]. Therefore, the late-onset progressive neuropathy, ataxia, and leukodystrophy in patients with ClpP mutations might be mediated by innate immune activation. Our novel observation that DNAJA3 shows significantly enriched interaction with disease proteins that are responsible for spinocerebellar ataxia, including the nuclear transcription modulator ATXN1 or the cytosolic stress response factor ATXN3, might be viewed in this context.

In contrast, the early-onset infertility typical of ClpP-mutant Perrault syndrome could be due to meiosis defects, where the handling of nuclear DNA is impaired in similar ways as the handling of mitochondrial nucleoids in ClpP cells. While the inflammatory tissue destruction is a joint feature of mutants in TFAM/ClpP/PINK1/PARKIN, only the ClpP loss-of-function results in selective massive affection of female and male meiosis. They show complete infertility with azoospermia, which is much more severe than the sperm motility impairment usually associated with bioenergetic respiratory or glycolysis failure [63]. Thus, there must be ClpP-specific effects that can be explained only extra-mitochondrially.

One potential mediator of retrograde signaling from the mitochondria to the nucleus seems to be the co-chaperone DNAJA3 that is mostly localized to the mitochondrial matrix, and particularly, the short isoform normally has minimal cytosolic retention time. Therefore, physiologically, only its long isoform would be predicted to interact in the cytosol with the JAK-STAT pathway [46]. The findings above seem paradoxical, since we observed the short isoform of DNAJA3 to accumulate in mitochondria and nucleus, while STAT1 abundance is further enhanced in the cytosol. However, a scenario is conceivable where excessive amounts of DNAJA3 short isoform interfere with the correct disassembly of nuclear transcription factor complexes, thus overactivating the *Stat1* gene transcription; in parallel, some compensatory efforts might retain STAT1 protein in the cytosol and minimize its unwanted overexpression. It is also possible that the overactivation of *Stat1* gene transcription is not downstream from DNAJA3; STAT1 was reported to repress mitochondrially encoded transcripts [36], so it might be activated in a primary response to modulate the elevated mtDNA dosage and altered mtRNA transcription of ClpP-null cells.

It is interesting to note a very recent report on the dysfunction of another mitochondrial DnaJ homolog known as DNAJC30. This factor appears to have a crucial role in the assembly/disassembly and turnover of the respiratory complex *N*-module. Its mutation results in a neurodegenerative process that selectively affects the optical nerve [68]. It has previously never been understood how different gene mutations that result in impairment of mitochondrial function would have vastly different tissue-specific consequences, if bioenergetics deficits are the only underlying problem. If differently tuned inflammatory consequences of each gene mutation are responsible for the long-term clinical consequences, then the tissue specificity variance of mitochondrial pathology may be much easier to understand.

## Conclusions

Overall, our investigation of ClpP-null mouse brains and MEFs detected UPR^mt^ anomalies prominently for DNAJA3. Enhanced levels and redistribution were also observed for its interactor protein STAT1, with induction of *Stat1* mRNA. Both DNAJA3 and STAT1 were altered in the intra-mitochondrial and extra-mitochondrial compartments, and their accumulations may underlie the transcriptional induction of downstream inflammatory factors. Instead of a generic activation of interferon-dependent innate immune pathways, a fine-tuning of cytosolic sensors and RLR signaling for maximal detection of dsDNA/dsRNA/ssRNA was observed, in good correlation to increased dosage of abnormal mtDNA and mitoribosomes in ClpP-null cells. These anomalies may be ClpP-specific, but perhaps similar data might also be found in other variants of Perrault syndrome. These DNAJA3 anomalies are followed by early-onset deafness in ClpP-mutant patients, while a recent report showed anomalies of the mitochondrial matrix chaperone DNAJC30 to occur in hereditary optic nerve atrophy. Therefore, the tissue-specific pattern of neurodegeneration in mitochondrial diseases could depend on the molecular profile of mitochondrial pathology and of immunological activation. Innate immunity pathways were similarly affected by ClpP homozygous deletion as in previous reports about TFAM heterozygous deletion, but more severe than findings in PINK1 and PARKIN mutants. Thus, the cytosolic sensors of DNA and RNA might constitute a general response network to dysfunctions in the mitochondria and in the cytosolic translation pathway. For all such disorders, the documentation of their innate immune profile and a neuroprotective trial via STING depletion may be rewarding in the future.

## Supplementary Material

Figure S1

Figure S2

Table S1
