# Supplementary figures and images for: Increased presence of nuclear DNAJA3 and upregulation of cytosolic STAT1 and of nucleic acid sensors trigger innate immunity in the ClpP-null mouse
Source: Neurogenetics. Author manuscript; Available in PMC 2021 Oct 1. (PMC8426249; doi:10.1007/s10048-021-00657-2)

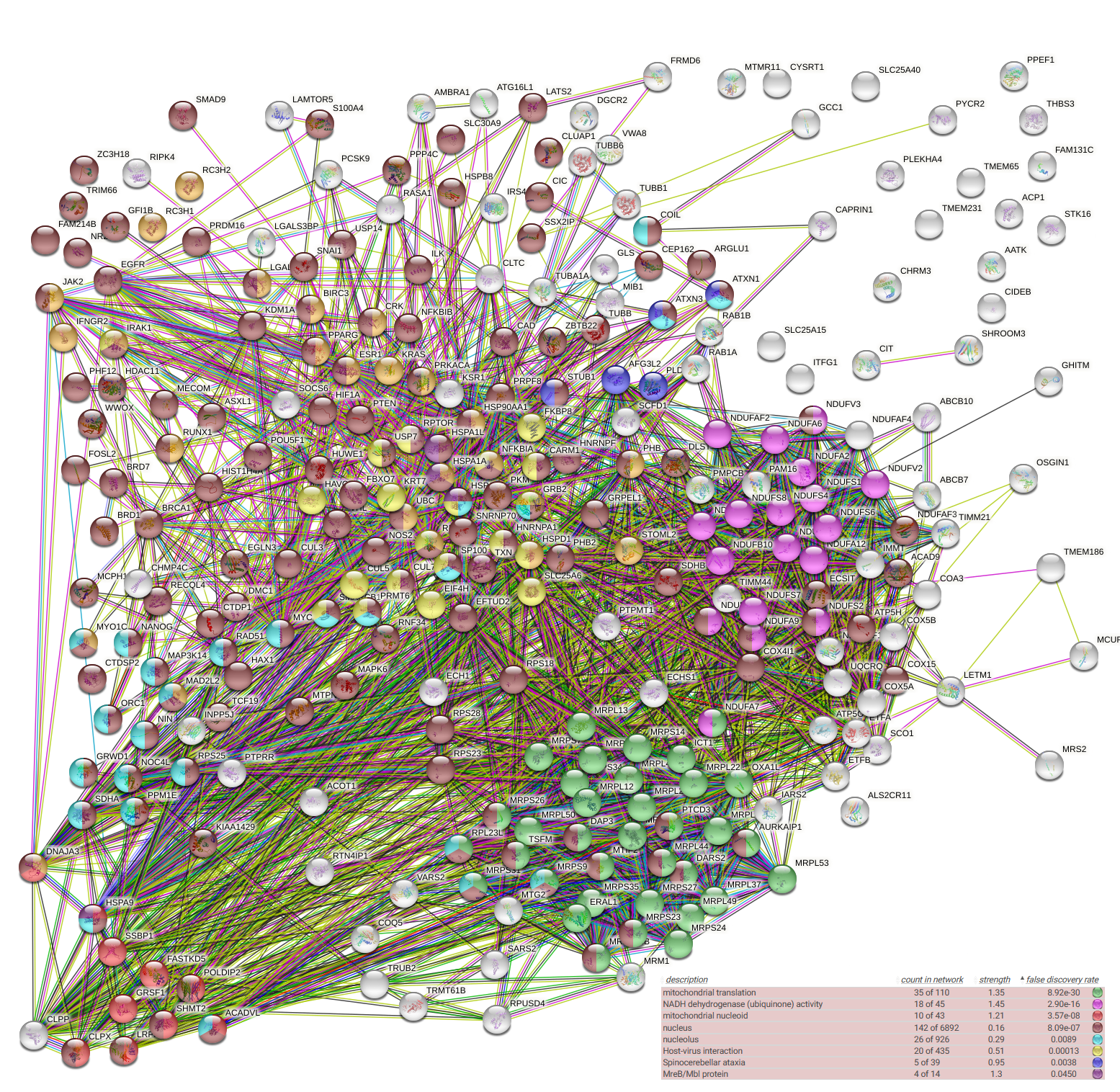

Supplement: Figure S1 [file NIHMS1739202-supplement-Figure_S1.tif]
